# Modeling the role of p53 pulses in DNA damage- induced cell death decision

**DOI:** 10.1186/1471-2105-10-190

**Published:** 2009-06-22

**Authors:** Tingzhe Sun, Chun Chen, Yuanyuan Wu, Shuai Zhang, Jun Cui, Pingping Shen

**Affiliations:** 1 State Key Laboratory of Pharmaceutical Biotechnology, School of Life Sciences, Nanjing University, Nanjing, 210093, PR China

## Abstract

**Background:**

The tumor suppressor p53 plays pivotal roles in tumorigenesis suppression. Although oscillations of p53 have been extensively studied, the mechanism of p53 pulses and their physiological roles in DNA damage response remain unclear.

**Results:**

To address these questions we presented an integrated model in which Ataxia-Telangiectasia Mutated (ATM) activation and p53 oscillation were incorporated with downstream apoptotic events, particularly the interplays between Bcl-2 family proteins. We first reproduced digital oscillation of p53 as the response of normal cells to DNA damage. Subsequent modeling in mutant cells showed that high basal DNA damage is a plausible cause for sustained p53 pulses observed in tumor cells. Further computational analyses indicated that p53-dependent PUMA accumulation and the PUMA-controlled Bax activation switch might play pivotal roles to count p53 pulses and thus decide the cell fate.

**Conclusion:**

The high levels of basal DNA damage are responsible for generating sustained pulses of p53 in the tumor cells. Meanwhile, the Bax activation switch can count p53 pulses through PUMA accumulation and transfer it into death signal. Our modeling provides a plausible mechanism about how cells generate and orchestrate p53 pulses to tip the balance between survival and death.

## Background

Biological networks are abstract representation of biological systems, which capture many of their essential characteristics [1]. Computational modeling of biological networks predominantly obtains insight into their systems behaviors. Special attention is paid to the dynamical networks of cell cycle transitions, circadian rhythms and apoptosis [2-4]. Apoptosis, which evolves in an all-or-none fashion, is a self-defense machinery to eliminate cells that are potentially dangerous [5]. The process of cell death decision concerns an integration of multiple malignant inputs. Once the decision has been made, this event is considered to be a 'point of no return'.

Apoptosis is a precisely regulated program in response to cellular stress. The tumor suppressor p53 plays essential roles in mediating apoptosis progress as evidenced by frequent mutations of p53 in tumors [6]. Activation of p53, which triggers a transcription regime, is a critical response in cell death decision [6]. Known targets for p53 in promoting apoptosis include PUMA, Noxa, Bid, Bax, as well as other death inducing factors, such as p53AIP, DR5, caspase-6, PERP and Fas [7]. Some negative regulators are also p53 transcriptional targets. A well documented p53-inducible protein, MDM2, targets p53 for proteosome degradation and keeps p53 at low levels in unstressed state [8].

Mitochondria play a crucial role in apoptosis by sensing external and/or internal apoptotic signals and responding by mitochondria outer membrane permeabilization (MOMP). MOMP is characterized by permeabilization pores formation and release of apoptotic factors such as cytochrome c, Smac/DIABLO and Omi/Htra 2 from mitochondria inter-membrane space, which will trigger caspase activation and initiate a serial downstream events to ensure apoptosis [9]. Although the exact mechanism of MOMP remains elusive, most experiments support that MOMP is governed by intricate interactions among Bcl-2 family members. Bcl-2 family members are composed of three functional groups: pro-apoptotic multi-domain proteins (Bax, Bak), anti-apoptotics (Bcl-2, BclxL, Bcl-w, A1, Mcl-1), and BH3-only proteins (e.g. PUMA, NOXA, Bid) [9,10]. In resting cells, Bax predominantly exists as soluble monomers in the cytosol, while monomeric Bak is inserted into mitochondria outer membrane [9]. In response to apoptotic stimuli, Bax monomers translocate from the cytosol to MOM, and both Bax and Bak oligomerize to form permeabilization pores which contribute to MOMP [9]. Anti-apoptotic members block cell death following numerous insults. Bcl-2, together with its anti-apoptotic group members, functions as potent apoptosis inhibitors by binding to Bax/Bak and BH3-only proteins to block their functions [9,10]. The third group terms 'BH3-only'. Selected BH3-only members termed 'Activator' are sufficient to trigger a conformational change and oligomerization of Bax/Bak, while other BH3-only members termed as 'enabler' can displace 'Activator' from the sequestration of anti-apoptotics [10].

MDM2 is p53-inducible and functions as a negative regulator of p53. This pattern confirms a negative feedback which is fundamental for p53 oscillations. Indeed, damped oscillations of p53 have been observed in cell populations [11]. Several mathematical models have been proposed to explain the damped oscillation over cell population. Bar-Or *et al*. presented a simplified model in an attempt to explain the mechanism of damped oscillation [11], and similar dynamics were also presented by Monk *et al*. when introduced a time delay [12]. In a field-breaking study of p53-MDM2 in individual cells, Alon and his coworkers found the expression of p53 follows a series of pulses and the mean period of the oscillations are relatively fixed while the mean number of pulses increase with increasing irradiation dose [13]. This is called 'digital oscillation'. The preeminent model explaining the digital behavior comes from Ma *et al*. [14]. They introduced a stochastic process in damage repair process to reproduce the digital pulses successfully. Ciliberto *et al*. [15] and Chickarmane *et al*. [16] manipulated the p53 system from stable steady state to a region of stable limit cycle in response to damage, which is then drawn back when damage is eliminated. Zhang *et al*. compared these models and delineated several new scenarios although the speculated mechanisms seemed to be cell type specific [17]. Alon and his coworkers also found that p53 performs sustained oscillation with γ-irradiation in tumor cell lines when observations lasted longer [18]. Batchelor *et al*. found a key mediator Wip1 in p53 signaling and consolidated p53 performs sustained oscillation [19]. Several models focused on deciphering this phenomenon. Puszynski *et al*. evaluated the oscillation and bistability in stochastic p53 system and proposed that deficiency in phosphatase and tensin homologue deleted on chromosome ten (PTEN) regulations contributes to the sustained oscillation [20]. Proctor *et al*. reconstructed two minimal models in p53 regulation and showed highly variable p53 pulses, however, one of their constructions referred to ARF pathway [21]. Although numerous models aimed to illustrate the digital or sustained pulses of p53 to fit experimental results, several questions remain elusive. First, how does the sustained p53 oscillation in tumor cell lines originates from the well studied digital p53 pulses? Second, what is the physiological role of p53 pulses on DNA damage? Similar questions raised by Tyson in a recent review showed aspirations for elucidating the linkage between digital and sustained oscillations and for the emergence of a counting mechanism of p53 pulses [22].

In the paper, we proposed a plausible model to clarify two key questions in this field: (1). How sustained oscillation of p53 originates from digital oscillations. (2). How cells count p53 pulses and make a decision between survival and death. Three modules of p53 network (ATM activation module, p53-MDM2 oscillation module and the Bax activation module) were interconnected. The interlinked positive feedbacks in ATM activation module confirm a bistable switch, which controls downstream p53-MDM2 module. The ATM switch can turn off the p53 pulses when damage is repaired and thus elicit digital p53 pulses. A mutant switch with high basal DNA damage, however, can never turn off the downstream oscillation. In addition, we proposed that the 'Bax activation switch' as described in our previous work can 'count' p53 pulses through accumulated PUMA and decide cell fate. Our modeling successfully provided a plausible mechanism of p53 pulses in governing DNA damage-induced cell death decision.

## Results

**Figure 1:**
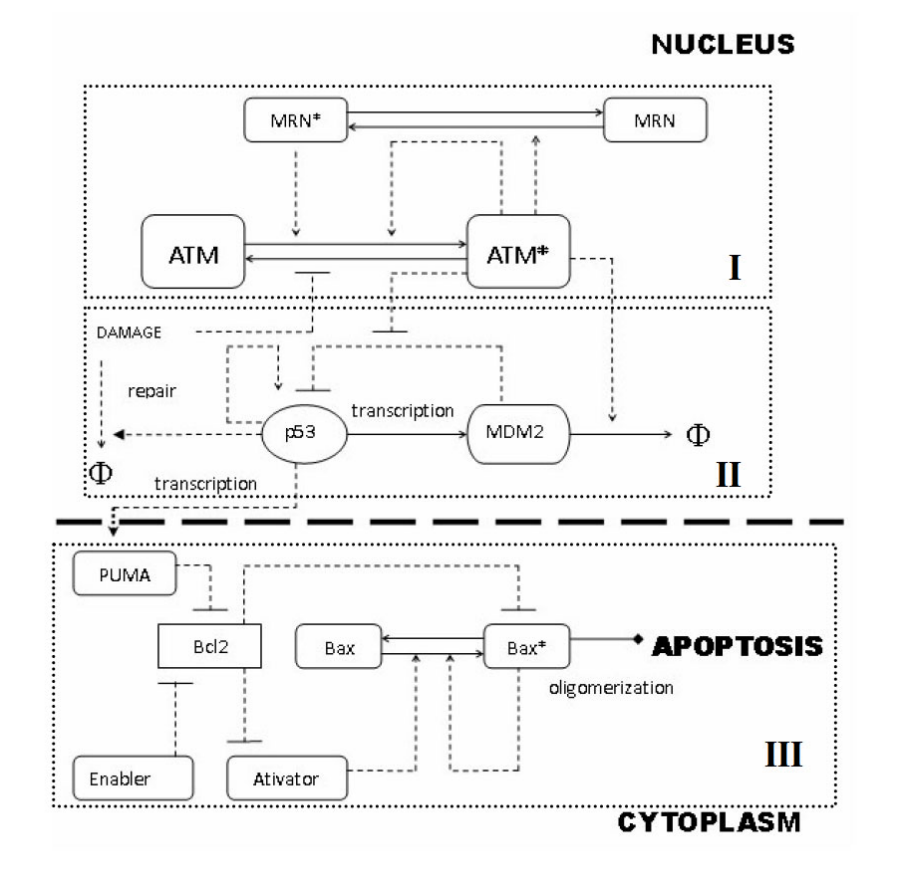
**Schematic Representation of the regulatory network**. Three modules are represented in corresponding dotted Box. I: ATM module. This module delineates ATM activation through both MRN mediated process and autophosphorylation. II: p53-MDM2 module. The tumor suppressor p53 participates in DNA breaks repair process in multiple ways and also induces PUMA expression which functions as 'input signals' to the downstream Bax activation switch. III: Bax activation switch module. Pro-apoptotic members of Bcl2 family termed 'BH3-only' proteins either dissociates Bax/Bak from the sequestration of anti-death members (enabler) or directly activates Bax/Bak (activator). PUMA serves as 'enabler' and is p53 inducible. PUMA functions as 'input' to the downstream Bax activation module. Dotted lines donate regulatory control and solid lines represent mass flow. Arrow means activation, horizontal bar means inhibition. 'Φ' donates degradation.

Our models are based on established biological facts and supplemented by some assumptions and simplifications. The schematic representation of p53 network is given in Figure 1. The details and necessary model simplifications are described in "Methods". The model can be further dissected into three functionally connected sub-modules, the interconnections of which are based on the input-out patterns (See Figure 1 for details). We then investigated the bifurcation properties of each function module.

### ATM activation switch (ATM module)

**Figure 2:**
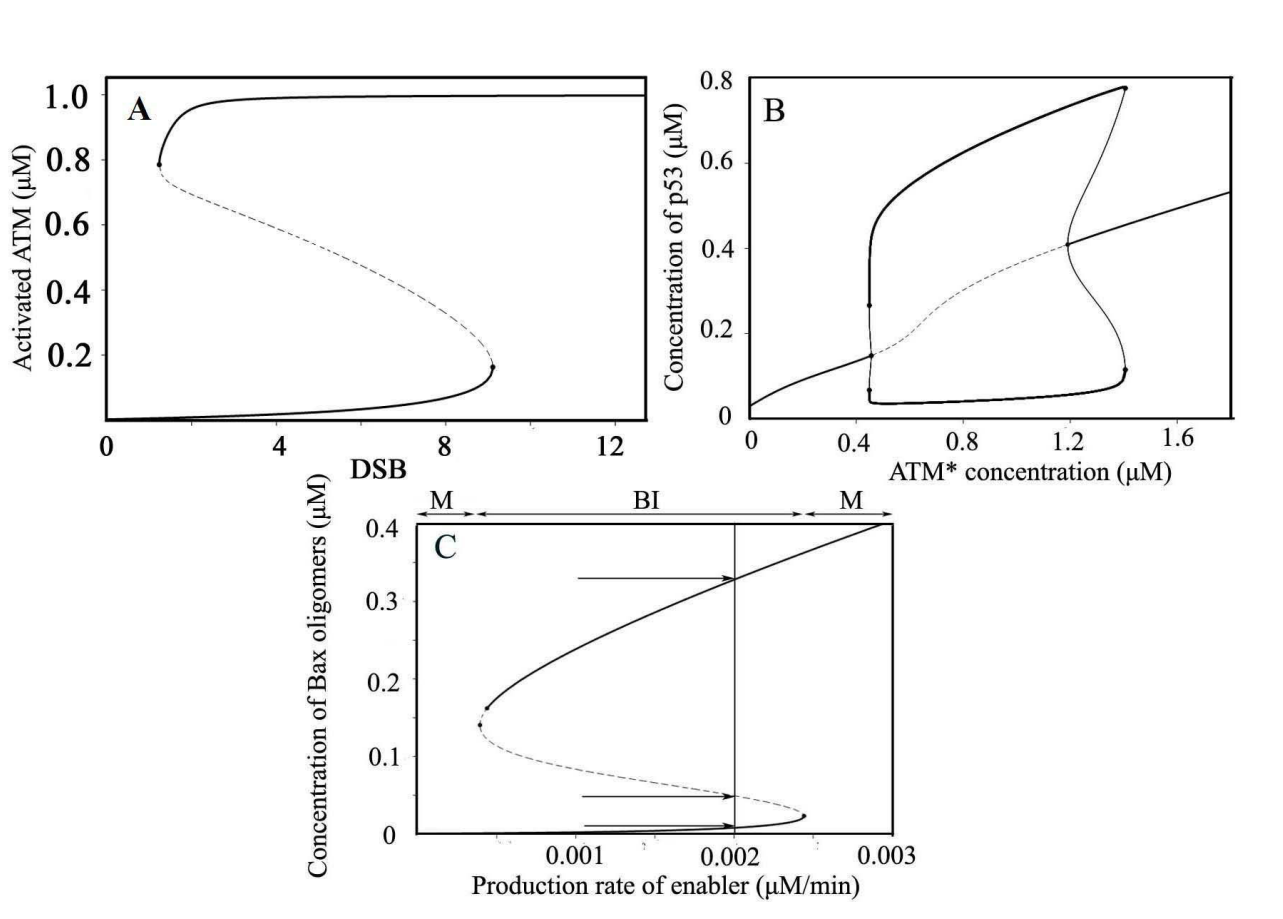
**Bifurcation diagram of three functional modules**. **A) **Bifurcation diagram of activated ATM (ATM*) (Limit points: 1.22 and 9.10). **B) **Bifurcation diagram for p53 (Hopf bifurcation points are 0.47 and 1.20). **C) **Bifurcation diagram of oligomerized Bax versus production rate of Enabler (saddle node points are 0.0004 and 0.0024). Arrows indicate steady state values (0.008, 0.050, 0.328 μM from bottom to top, M: monostable. BI: bistable).

We represented how ATM shows bistability and transmits damage signals. Figure 2A depicts the bifurcation diagram. A sub-threshold damage (< 9.10, the right limit point) only activates minimal fractions of ATM. Once damage is repaired, activated ATM rapidly drops to low levels. When damage overrides the threshold level, activated ATM rapidly jumps to a high level and finally becomes fully activated (> 99.8%). The levels of activated ATM will not fall until the remaining double strand breaks (DSBs) visits the limit point (DSB = 1.22). The solid curve indicates stable steady state, while the dashed line means unstable steady state. Bistability primarily comes from positive feedback. In our model, ATM is subject to two positive feedback loops: one originates from interactions with Mre11-Rad50-Nbs1 (MRN) complex and the other from auto-activation (intermolecular autophosphorylation), which primarily lead to bistability. Cooperation of them quickens the transition from 'off' to 'on' state [23], ensuring accelerated upswing of activated ATM levels. Experimental results indicated an abruptly onset of activated ATM and reaches saturation within an hour or minutes [24,25], which is consistent with our simulation results. Our results above suggest the interconnected positive feedback ensures bistability for ATM.

### p53-MDM2 interaction module

We next proceed to analyze the p53-MDM2 module. In unstressed cells, degradation of p53 is rapid, while the degradation of MDM2 is slow (δ_MDM2 _= 0.002, k_atm_≈0, k_atm_: ATM-induced accelerated degradation rate of MDM2). It keeps p53 at low levels and no oscillation is shown (see Figure 2B, for ATM* = 0.0005 and data not shown). A super-threshold damage (set [ATM*] = 1) induces a reduced MDM2 dependent degradation of p53 (k_md _= 0.007, set [ATM*] = 1) and accelerated MDM2 auto-degradation (k_atm _= 0.003). Both parameters (k_md _and k_atm_) are controlled by activated ATM ([ATM*]) levels. Previous work supports this assumption [26]. The dynamics of p53 is largely influenced by ATM status, while the abrupt onset of activated ATM quickly moves the p53-MDM2 system from stable steady state to stable limit cycle and draws p53 into the oscillatory region (Figure 2B). When the level of activated ATM decreases, p53 level reverts to its original steady state and the oscillation of p53 stops. The bifurcation parameter was chosen to be [ATM*] in consideration of connecting the p53-MDM2 module with the upstream ATM module. These results suggest that ATM activation status can prominently influence on dynamics of the p53-MDM2 module.

### Bax activation module

Bistable behavior in Bax module was described in our previous works [27,28]. Here illustrates the bifurcation diagram (Figure 2C). The solid curves indicate stable steady state, while the dashed line characterizes the loci of unstable steady state leading to either low or high steady state on slight perturbations. As described above, the disruption of mitochondria outer membrane is largely attributed to the levels of Bax oligomers, and an abrupt upswing of Bax oligomers will definitely lead to apoptosis. Therefore, the dashed line also defines the threshold stimuli to trigger apoptosis. In the mono-stable region, however, no perturbation has effects on the steady state level of Bax oligomers and leads to either monotone survival or death.

The results from individual modules above clearly showed oscillatory or bistable behavior in p53 related network. We then investigated the integrated behavior of these modules.

### Digital or sustained oscillation

**Figure 3:**
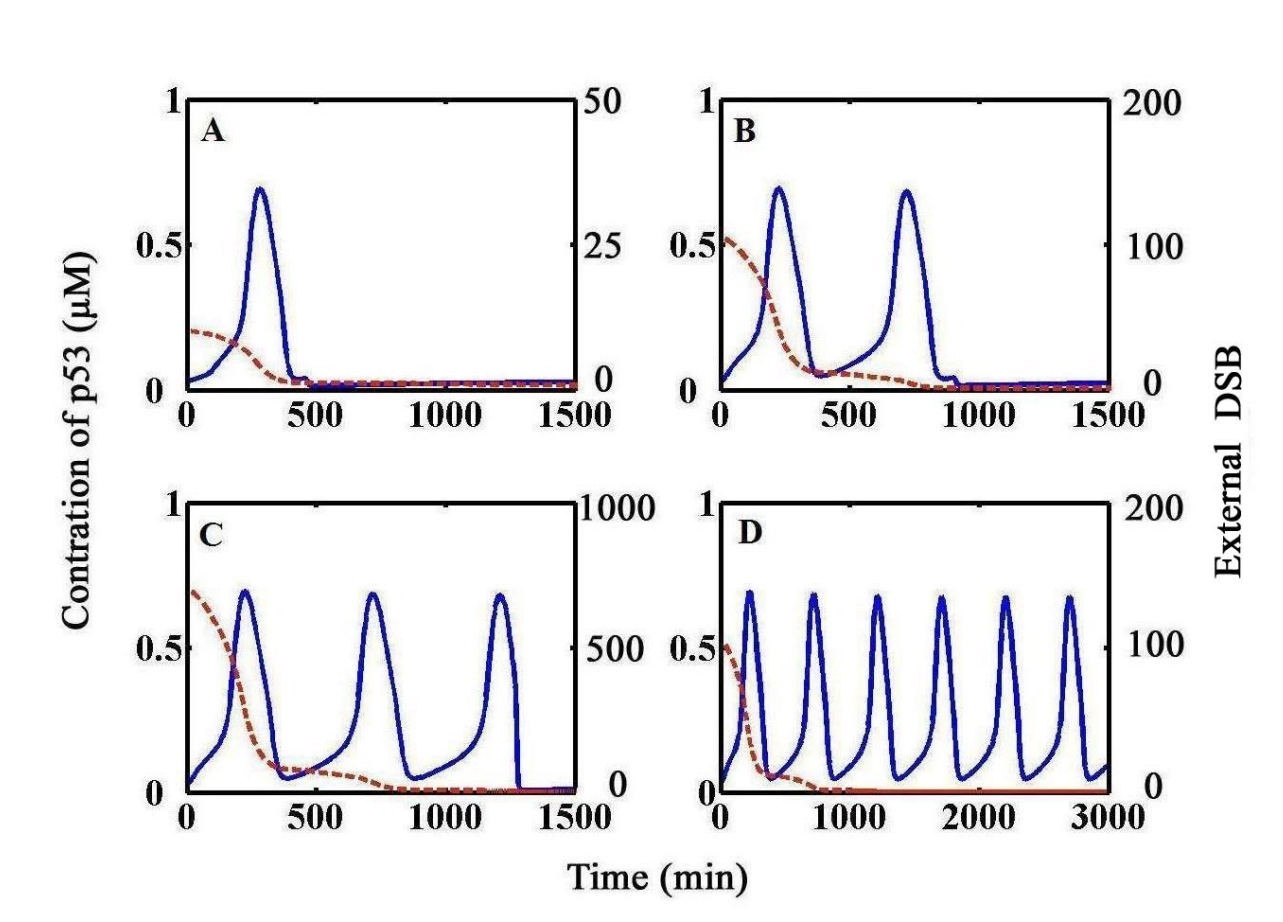
**Temporal responses of p53 in normal cells and tumor cells**. Time series plot of p53 (solid blue line) and repair process (dotted red line) at various initial damage levels (A-C: normal cells, k_5 _= 1, D: tumor cells, k_5 _= 3). **A) **IR = 0.3 Gy. **B) **IR = 3 Gy. **C) **IR = 20 Gy. **D) **IR = 3 Gy. The dynamics of p53 shows digital or undamped oscillations.

We presented the time course evolution of p53 in response to distinct external damages. Figure 3 shows the time series of simulation results about p53 and damage levels. p53 always remains at the steady state when no stimulus is experienced (data not shown). Note that DNA damage we discussed here can be divided into two categories: one is basal DSB (or DNA break) and the other is external DSB. Basal level of DSBs indicates that even in non-stressed cells, it definitely exists due to mitosis and/or meiosis although differs in levels and cannot be fully repaired; external DSBs are induced by external stimuli (e.g. γ-irradiation) in stressed cells and can be fully repaired. Non-zero irradiation dose elicits a digital oscillatory of p53 (Figure 3A–C, IR = 0.3 Gy, 3 Gy, 20 Gy respectively). During each p53 pulse, external DSBs are repaired until they fully drop down (Figure 3A–C, dotted line). Upstream events such as ATM activation, MDM2 transcription, MDM2-dependent p53 degradation and subsequent MDM2 destabilization primarily contribute to the rise and fall of p53, which is not described in details.

Computational analysis showed that p53 oscillates persistently when the system harbors basal levels of unrepaired DSBs (Figure 3D, here we set k_5 _= 3, k_5 _donates basal DSB levels). In Figure 3D, irradiation induced DSBs are completely repaired (dotted curve) but p53 still oscillates. The oscillation evolves with relatively fixed amplitude and period. It possibly implies that there must exist certain mechanism to 'memorize' the effect of initial external damage.

**Figure 4:**
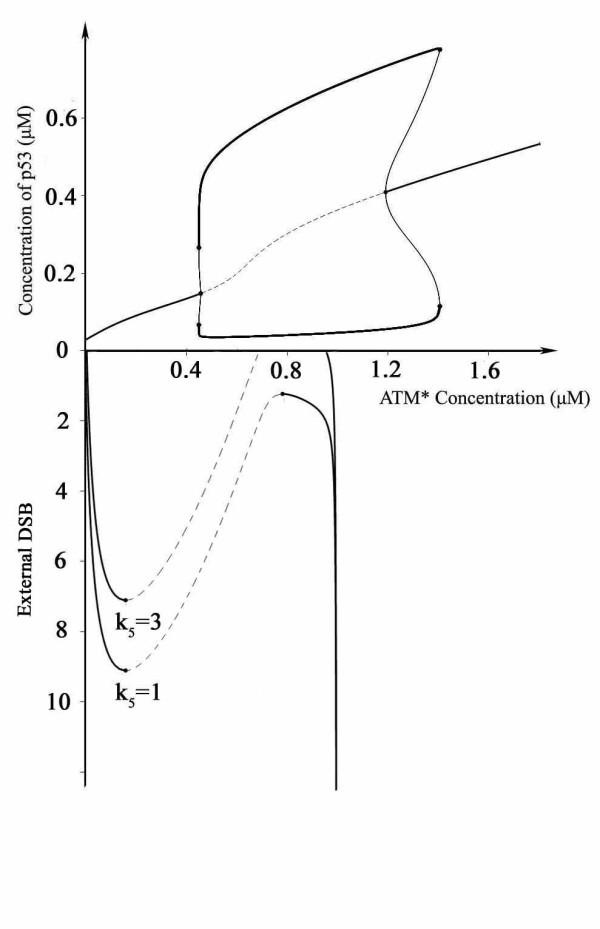
**Schematic illustration of the mechanism of digital and sustained oscillation**. The upper curve is a bifurcation diagram, p53 versus concentration of activated ATM. The lower curve is the dependence of activated ATM on the damage levels. The parameter k_5 _donates basal unrepaired DNA damage ([ATM*]: activated ATM. The limit point for the mutant switch is 7.11, for non-mutant switch 9.10).

We further investigated the underlying mechanism that discriminates the sustained oscillation from the digital oscillation (Figure 4). The parameter k_5 _donates basal level of DSBs. We set k_5 _= 1 which highlights a physiological damage, primarily because even in the absence of external stimuli, there appear to be constant, significant levels of physiological DNA damage [29]. Higher assigned values correspond to pathological circumstances (e.g. tumor cells) in our simulations. In physiological conditions (k_5 _= 1), once DSB levels transcend the threshold (DSB = 9.10), ATM* dramatically flips from low to high state and rapidly becomes fully activated. As DSB is repaired, the levels of ATM* slowly decrease, while an abrupt decline occurs when DSB level falls below the limit point (DSB = 1.22), and then the switch turns off. The duration of high levels of ATM*, which is indicative of the duration of damage repair process, determines the duration time that p53 spends in the oscillatory region. A longer duration in the oscillatory region corresponds to more DSBs and more p53 pulses. This phenomenon finally ensures the digital oscillations in context of different external stimuli. In pathological conditions, however, the ATM functionally becomes a one-way switch (see Figure 4, k_5 _= 3). An external super-threshold irradiation dose (DSB > 7.11) results in the elevation of ATM* level and turns on the switch. Once fully activated, ATM* never falls down albeit external DSBs are fully repaired (See Figure 3D). The sustained high levels of ATM* 'tell' p53 to oscillate permanently and p53 accepts that idea with no doubt because it has limited knowledge of what is happening on upstream breaks repair process. This ensures a sustained p53 oscillation. Similar results also emerged in other cases (data not shown). Experimental results by Olivier *et al*. have proved that some tumor cell lines have defective or relaxed checkpoint control and tolerate unrepaired DNA lesions[30]. Overall, these results suggest that a physiologically low level of basal DNA damage defines a hysteretic response of ATM* and digital oscillations of p53, while in pathological conditions, ATM activation becomes a one-way switch and cannot revert to its initial level once activated. These two cases are determined by the basal DSBs. Experiments by Geva-Zatorsky *et al*. are based on MCF7 breast cancer cells and the observation that p53 performs sustained oscillation is probably ascribed to the significant levels of basal DNA lesions in tumor cell lines[18]. Our assumptions are consistent with experimental results.

### Bax activation switch: counts and decides

**Figure 5:**
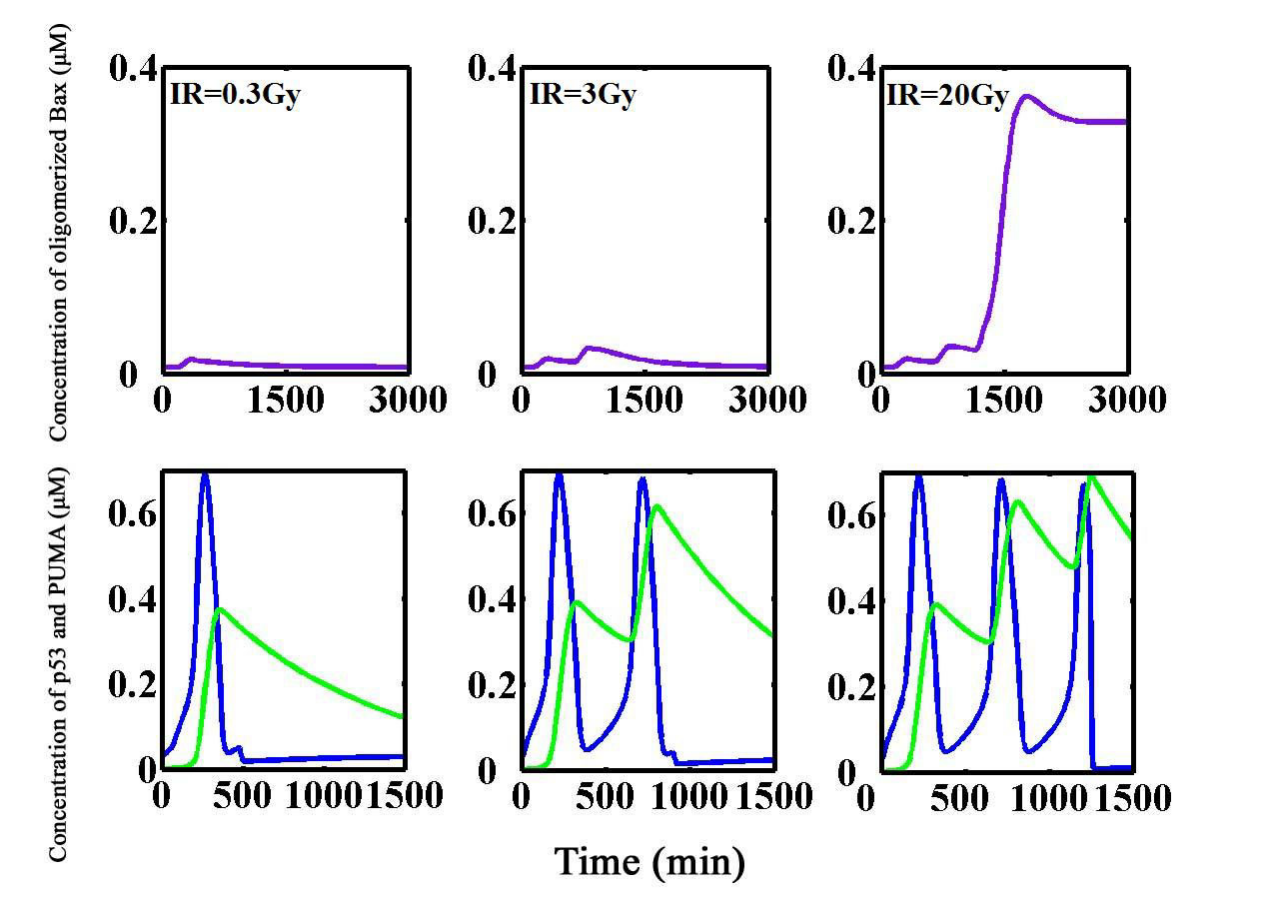
**Dynamics of the integrated model**. Upper panel: Time course plot of oligomerized Bax (violet). Lower panel: Time course plot of p53 (blue) and PUMA (green). PUMA levels are gradually accumulated, ratcheting up at each p53 pulse until it reaches a threshold level at which the Bax activation switch turns on (IR = 0.3, 3, and 20 Gy respectively).

Previous work by our group showed that the Bax activation switch, which computes multiple apoptotic inputs into an all-or-none fashion corresponds to either survival or death [27,28]. However, what signals register DNA damage and then impinge on downstream apoptosis remains unsolved. Here we provided a plausible mechanism by our mathematical simulations (Figure 5). On the left panel (IR = 0.3 Gy), a slight irradiation dose triggers one p53 pulse and the external DSBs are rapidly repaired. Expression level of PUMA increases as p53 becomes activated with a delay, which indicates the time for transcription, translocation and translation. Newly produced PUMA brings perturbation to downstream Bax activation switch and a subtle elevation in Bax oligomers appears but soon tarnishes. Mild perturbation is tolerated, and all components return to their original steady state hours later. A ten-fold irradiation dose (IR = 3 Gy, Figure 5, middle panel) evokes two p53 pulses followed by a step-wise raise in PUMA. The damage is also repaired and Bax oligomers eventually revert to the initial steady state (0.008 μM). In this case, Bax oligomers reach a higher peak value compared with the one under lower irradiation. Also, it takes a longer time for Bax oligomers to return to resting state. A more severe irradiation (IR = 20 gy, row 2 column 3) evokes three pulses, and Bax oligomers have an abrupt onset and finally jump to a high level (0.328 μM). PUMA undergoes a step-wise upswing during each pulse of p53 (Figure 5, right panel).

**Figure 6:**
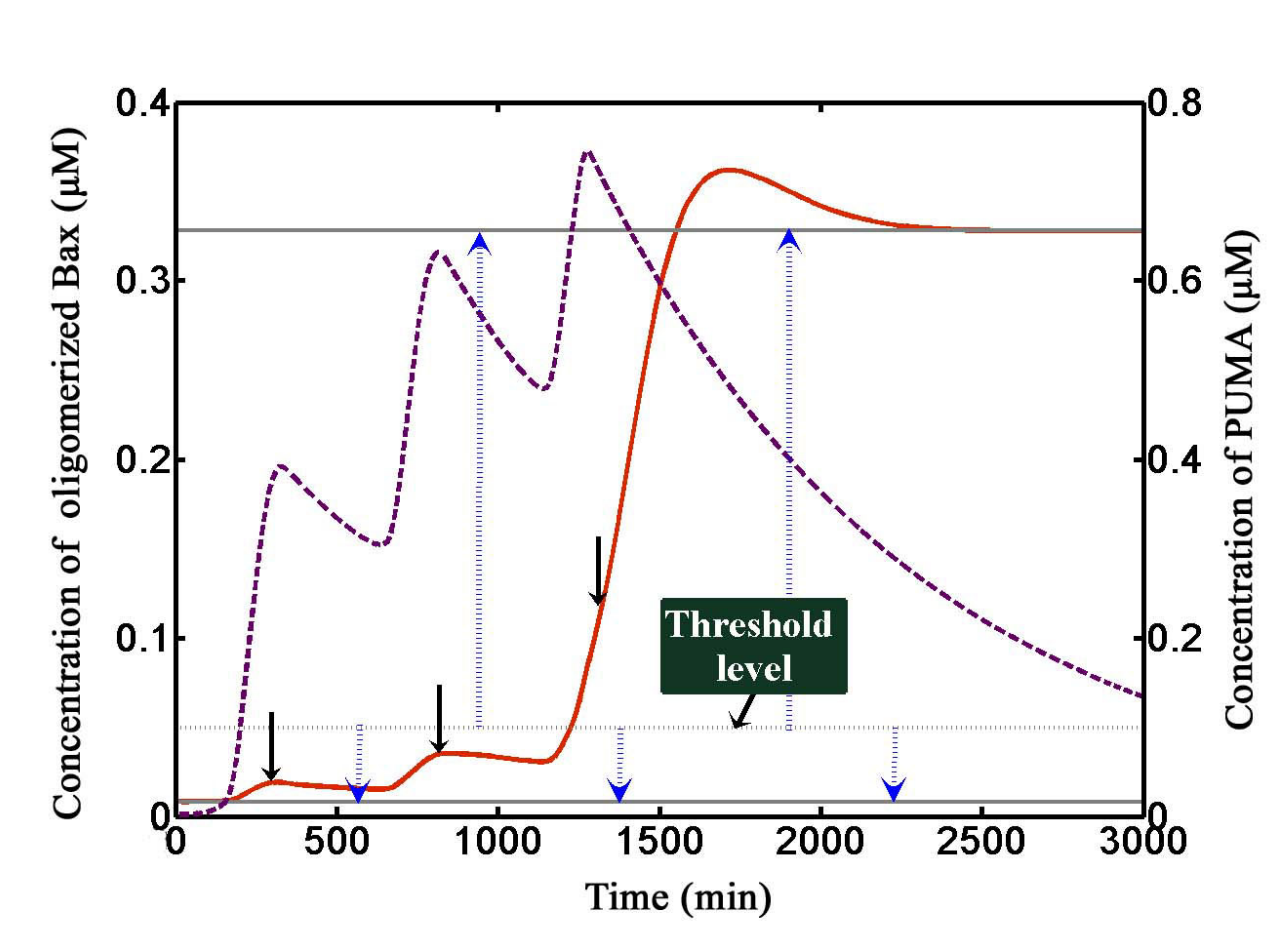
**Illustration of the counting mechanism**. The steady state is shown. (solid grey: stable steady state; dotted grey: unstable steady state. p4 = 0.002). The corresponding values of the steady state are shown as indicated in Figure 2C (0.008, 0.050, 0.328 μM from bottom to top). Dotted blue arrows indicate the equilibrium concentrations. The threshold level is indicated at the text box. Solid black arrows represent the Bax concentration reached during each pulse of p53. Temporal responses of oligomerized Bax (solid red) and PUMA (dashed violet) are shown.

Then we further illustrated the underlying counting mechanism (Figure 6, for IR = 20 Gy). The steady state values of Bax were indicated by arrows in Figure 2C (Three steady states in Figure 2C indicated by arrows correspond to dark grey lines in Figure 6, where solid lines mean stable steady state and dotted line means unstable steady state). Dotted blue arrows show directions of evolution. In the first p53 pulse, transcription of PUMA quickly begins. The level of PUMA rises and falls, which we called it a 'PUMA boosting'. The 'Enabler' PUMA is sensed by Bax activation switch. The new-coming PUMA perturbs Bax activation switch and dissociates 'Activator' (e.g. Bid) from the sequestration of Bcl-2 (and/or other Bcl-2 anti-apoptotics) and then these liberated 'Activator' directly activate Bax. Activated Bax then oligomerizes and contributes to elevated levels of Bax oligomers. When degradation rate of PUMA overrides the production owing to reduced transcription, the levels of PUMA decay. PUMA is then neutralized by newly synthesized Bcl-2 (and/or other anti-apoptotics). In turn Bax oligomers decrease due to degradation and oligomers dissociation. The level of Bax oligomers is a bit higher ([Bax_olig_] = 0.019 μM) than the original steady state (0.008 μM) at the time when a second PUMA boosting is encountered. Bax oligomers is accumulated but still below the threshold ([Bax_olig_] = 0.035 μM) at the end of the second PUMA boosting. A third p53 pulse evokes a third PUMA boosting and Bax oligomers further accumulate to surmount the threshold. Once Bax oligomers are above the threshold, they will not decrease and stay at the high steady state. Bax oligomers disrupt mitochondria outer membrane and unleash cytochrome c, which then leads the activation of caspase-3 ensuing apoptosis. Thus, the information on DNA damage is successfully transferred to the Bax activation switch, and the cell death decision has been made irreversibly.

Taken together, this model delineated a clear picture from damage sensor to downstream decision making by Bax activation switch. In response to irradiation, ATM becomes phosphorylated and fully activated. Activated ATM then phosphorylates multiple substrates to trigger p53 pulses. The external DSBs are repaired during each p53 pulse until they fall below the threshold. Elimination of DSBs quickly shuts down the ATM switch and concomitant p53 oscillations, thus ensures digital pulses. During each p53 pulse, PUMA accumulates in a step-wise manner. PUMA perturbs downstream Bax activation switch. If the perturbation is strong enough, the threshold level will be overridden with an abrupt onset of Bax oligomers which finally contribute to apoptosis. Counting finally comes true: one or two pulses lead to survival and three pulses to death (Note that we do not restrict our assumption that only three pulses contribute to death, but suggest that the number of pulses required to trigger apoptosis may differ in different cell types). The experimental observations by Yu *et al*. confirmed a direct p53-PUMA-Bax interconnection as PUMA or Bax knockout cells become defective in commitment to apoptosis although p53 is transfected [31]. A plausible explanation of this experimental results can be derived from our model in which p53-induced PUMA expression is essential for 'counting' and death decision and cells deficient in either downstream component (Bax or PUMA) cannot get access to apoptosis. In all, our modeling results suggest that the 'Bax activation switch' can 'count' the p53 pulses through accumulated PUMA and plays a pivotal role in death decision process.

## Discussion

The present study gave insight into the oscillatory dynamics of p53 and demonstrates that how cells show digital or sustained oscillations in the context of different basal DNA damage. We further provided a plausible mechanism about how cells count the p53 pulses in DNA damage-induced cell death decision.

We incorporated essential aspects of ATM activation and p53 oscillator modules. Simulation results showed that p53 oscillates in a digital manner. Further computational analysis in mutant phonotype came to a new oscillation pattern. Observations by Geva-Zatorsky *et al*. indicated that tumor cells, under specific circumstances perform sustained oscillation [18]. We obtained insights into this dynamics by assigning k_5 _a larger value (k_5 _= 3) indicative of significant levels of basal unrepaired DNA breaks and got an exciting observation that p53 shows sustained oscillation with superthreshold stimuli in these cells (Figure 3D). In tumor cells, a considerable amount of unrepaired DNA breaks was verified [29,30], and therefore we suggest that higher levels of basal DNA breaks in tumor cells would result in sustained oscillation of p53. The discrimination of sustained and digital oscillation patterns can be ascribed to the status of ATM switch, some of which define a one-way switch with high levels of basal DNA damage (Figure 4). Unlike normal cells, tumor cells often show significant activation of ATM and ATM dependent check point pathways under non-stressed conditions [32-35]. On the other hand, deactivation (dephosphorylation) of ATM is regulated by PP2A, which is an important phosphatase in cells [36]. Meanwhile, PP2A subunit mutations are found in a broad set of human cancers and most of these mutants are functionally defective [37,38]. Other phosphatases also contribute to the inactivation of ATM such as PP2C [39]. We speculated that many phosphatases can inactivate ATM when DNA damage repair progresses, but functional mutations of phosphatases ubiquitously found in the tumor cells (e.g. PP2A mutation) might reduce the inactivation efficiency of ATM (as the third term in Eq.1 in mutant ATM switch). So even after irradiation induced DNA breaks are repaired (probably corresponding to the non-irradiation conditions, which means the situation that external irradiation induced DNA breaks are fully repaired corresponds to the situation that no irradiation or irradiation induced DNA damage are encountered), the ATM switch can not be shut down and preserves highly activated state to ensure undamped pulses of p53 in the tumor cells.

Subsequent integration of p53 oscillator with Bax activation switch finally unveiled subtle control in cell death decision that two pulses or less lead to survival and three pulses to death. A key player in the signaling process is PUMA (other factors can also be included, see below and methods) which accumulates during each p53 pulse, perturbs the Bax activation switch and triggers apoptosis if threshold is overridden. Transcription of PUMA definitely provides a genuine gauge for the severity of DNA damage levels. With the help of PUMA (and also other p53 inducible factors), p53 can tip the balance between survival and death in a pulse-like manner.

Previous mathematical models proposed for p53 oscillation mainly concentrated on the reproduction of oscillation, but only a modicum of attention has been paid to the connections between p53 and death decision. Wee *et al*. strived for bridging the gap but they mainly referred to Akt/PKB pathways [40]. Zhang *et al*. [17] shed shimmering light in this field by discriminating p53 as three functionally different subpopulations. However the assumption needs further investigation. Puszynski *et al*. introduced a positive feedback between p53 and DNA breaks via an intermediate apoptotic protein and intended to decipher death decision process [20], but this positive feedback remains obscure. We developed an integrated model with great benefit to a full-scale understanding of the decision-making mechanism. We suggest that the Bax activation switch 'counts' p53 pulses through PUMA, and decides whether it be survival or death. p53, together with its transcriptional regime lies in the nexus of the upstream DNA damage sensor and the downstream Bax activation switch. It is indubitably through the p53 pulses that cells 'know' what happens in the nucleus (e.g. DNA integration status) and 'decides' what to do. Also, a paradox stems from the question: why do tumor cells that perform sustained oscillation escape from commitment to death? We proposed that tumor cell lines have defects in apoptosis execution [30,41-45], although p53 oscillates and PUMA accumulates. Perhaps only in tumor cells but not normal cells can sustained oscillations of p53 occur. Further experimental verifications are strongly demanded to clarify these modeling-based questions.

In this study of the p53 network, there is considerable interest in the dynamics of the system in cell fate decision. So a reduced representation of modules is convenient for analysis. Firstly, we took a simplistic representation to reproduce the p53 digital pulses. Noticeably, other proposed models are also applicable or closer to the exact mechanism for p53 oscillation [14-21]. Secondly, Batchelor *et al*. [19] discovered a key component Wip1 in p53 signaling which forms a feedback control over upstream ATM module. But the experiment was performed in tumor cell lines (MCF7), where p53 displays sustained oscillations. Besides they used persistent γ-irradiation experiments but not a pulse of γ-irradiation which was used by Geva-Zatorsky *et al*. [18]. Here we did not take Wip1 for consideration for two reasons: simplification and reproduction of digital p53 pulse (Note that it shows sustained pulses in the experiments according to Batchelor *et al*. [19]). In addition, most parameters that appear in our model derived from qualitative estimation to get the idiosyncrasies of network dynamics because their values are far from assured in experiments. A qualitative model provides insight into how cell behaves the way it does and how that behavior dependents on parameter fluctuations. Finally, downstream machineries other than the Bax activation switch that fulfill their roles through counting p53 pulses cannot be ruled out simply owing to the fact that in some cases PUMA^-/- ^cells are resistant to stimuli while Bax^-/- ^cells are not [46]. Furthermore, executioner caspase-6 is also identified as a transcriptional target of p53 [47], establishing a link between p53 and caspase apoptotic switch. Therefore, we proposed a general mechanism that damage signal is transmitted encoded by p53 through whatever transcripts and then downstream machineries, for instance, the Bax activation switch, counts and decides.

## Conclusion

Despite limitations and assumptions, our model first paves the way to an understanding of the counting mechanism and the role of p53 pulses in cell fate decision. Our model also bridges the gap between digital oscillation (physiological condition) and sustained oscillation (pathological condition). We hope that an impeccable appreciation of the intricate regulation of p53 network will help us to develop beneficial strategies for pharmaceutical and therapeutical purpose in the future.

## Methods

### Model and experimental basis

Reactions are interpreted into ODEs, simplified without sacrificing the fundamental dynamics of the network (For parameter values of ATM and p53-MDM2 modules see Table 1). ODE model can be also dissected into three functional modules as described in the text:

**Table 1:** Parameter values for ATM and p53 module

**ATM module**	**p53-MDM2 module**
k_1 _= 1 min^-1^	k_atm_' = 0.01 s^-1^
k_2 _= 0.01 μM	k_md_' = 0.03 min^-1^
k_3 _= 0.005 min^-1^	δ_p53 _= 0.01 min^-1^
k_4 _= 2.5 min^-1^	δ_MDM2 _= 0.002 min^-1^
k_5 _= 1	K_a _= 0.3 μM
k_6 _= 0.1 min^-1^	K_b _= 0.3 μM
k_7 _= 0.5 μM	K_c _= 0.3 μM
k_8 _= 1 min^-1^	K_d _= 0.5 μM
k_9 _= 0.01 μM	K_e _= 0.5 μM
k_10 _= 0.005 min^-1^	k_ind _= 0.02 μM·min^-1^
k_11 _= 0.8 μM·min^-1^	k_trans _= 0.1 μM·min^-1^
k_12 _= 0.1 μM	μ_p53 _= 0.003 μM·min^-1^
k_13 _= 0.02 μM^-1^·min^-1^	μ_MDM2 _= 0.002 μM·min^-1^
[ATM]_total _= 1 μM	m = 4
[MRN]_total _= 1 μM	n = 3 np = 3

### ATM activation and damage repair module

Dimeric or high-order multimeric ATM kinase is held inactive ([ATM]) in unirradiated cells and becomes activated ([ATM*]) through auto-phosphorylation [24]. ATM dimer dissociation and autophosphorylation rapidly initiate ATM kinase activity [24,25]. Experimental results highlighted that MRN complex can also activate ATM through phosphorylation. Lee *et al*. elucidated that ATM can be directly activated by MRN complex [48]. Experiments by Difilippantonio *et al*. supported an amplifying model: The MRN complex is recruited to sites of DNA damage on irradiation [49,50]. Following recruitment of MRN complex to these sites, it subsequently recruits and activates ATM. Activated ATM then phosphorylates histone H2AX (λH2AX), creating a platform for subsequent Nbs1 binding (an integral subunit of MRN complex), and causing additional MRN complexes attached to form an amplifying loop. Besides, experimental data also raised possibilities of ATM activation by other forms of DNA damage [51]. Activated ATM then phosphorylates p53, MDM2 and other proteins [25,52]. Based on these facts, we constructed equations 1–3.

#### ATM activation and deactivation

The first term represents the direct phosphorylation and activation of ATM by MRN complex. The third term in Eq.1 represents the ATM* deactivation rate. The irradiation induces dissociation of ATM from protein phosphatase 2A (PP2A) which dephosphorylates ATM to inhibit its kinase activity and loss of the associated protein phosphatase activity [36], for simplicity, we assumed that ATM inactivation rate decreases with DNA damage. The last term indicates autophosphorylation.

(1)

#### MRN complex activation and deactivation

The first term represents the ATM dependent activation of MRN.

(2)

#### DNA damage repair

The damage repair process depends on p53 concentration because p53 mediates almost all the five DNA repair processes [53] and we adopted a simplified representation. We assumed that 1 Gy causes 35 DSBs in deterministic equations [51], and the variable [Dam] corresponds to DSB levels. The right hand side of Eq.3 is further multiplied by [Dam] (DNA damage level) to exhibit an exponential-like decay to mimic the realistic dynamics [54].

(3)

Meanwhile, experimental results suggest that recruited MRN complex also contributes to the breaks repair [50] but we found little difference in the dynamics if the right hand side of Eq.3 was additionally multiplied by [MRN*] (see Additional file 1, Figure S1), and therefore the reduced model was accepted. The turnover of ATM and MRN is beyond our discussion, and total amount of these two proteins is taken as a constant value for simplicity ([ATM*] + [ATM] = [ATM]_total _= 1 μM, [MRN] + [MRN*] = [MRN]_total _= 1 μM. [MRN*]: DNA attached MRN, [MRN]: free MRN).

### p53-MDM2 feedback module

Based on experimental data, essential respects of the ODEs are described below.

#### p53 activation and degradation

Effects of p53 self-induction are considered in the model in accordance with recent research by Wang *et al*. [55]. An earlier work by Deffie *et al*. indicates that p53 promoter is also responsive to p53 regulation although undetected in direct binding [56]. These are two pieces of direct evidence that p53 is self-inducible. In addition, there are at least three positive feedback loops in p53 regulatory network [57], and positive feedback loops can be envisioned as 'self-induction', which means one species has positive correlation with itself. We incorporated all the information into one item with a Hill function representative of the nonlinear positive correlations for simplification. The third term donates the basal p53 degradation [58]. The fourth term represents the MDM2 dependent degradation of p53.

(4)

(5)

#### MDM2 induction and degradation

MDM2 also undergoes ATM dependent phosphorylation and phosphorylated MDM2 dissociates from the MDM2-p53 complex leading to reduced p53 degradation and accelerated auto-degradation under stress [26,59], the efficiency of MDM2 dependent degradation of p53 is reversely correlated with activated ATM. Hereby, k_md _(catalytic constant for MDM2 dependent degradation of p53) is reversely proportional to the activated ATM levels). ATM-dependent destabilization and auto-degradation of MDM2 is represented according to Ma *et al*. [14].

(6)

(7)

### PUMA induction and the Bax activation switch

In our previous model, we have illustrated that two independent positive feedback loops contribute to the bistable behavior of Bax activation which governs the mitochondria apoptosis pathways [27,28]. Once superthreshold stimuli are encountered in our simulations, an abrupt elevation of Bax oligomers will definitely disrupt the mitochondria and initiate apoptosis. Although the dynamical property of the Bax activation switch has been delineated, the cooperation of this switch with upstream signaling module remains to be determined. Here, we further investigated the cooperation of the p53 and the Bax activation switch (see Additional file 2, Table. S1 and our previous work [28]). PUMA is a transcriptional target of p53 and participates in the Bax activation switch as 'Enabler'. Note that the item for p53-induced expression of PUMA takes a form *k*_*puma*_·[*p*53]^*np*^/(*K*_*f*_^*np *^+ [*p*53]^*np*^) (Eq.14, see Additional file 2, Table. S1) but not as increased basal production rate of 'Enabler' (*p*_4_), primarily because PUMA functions as inputs to the downstream Bax activation switch, and this mathematical treatment creates a clear input-output pattern between p53-MDM2 module and downstream Bax activation module. Several issues are discussed below to clarify model simplification process. Notice that only PUMA appears in the equations. But as we know, Noxa is also induced by p53 in response to stimuli [6]. These two proteins are sometimes functionally redundant as 'Enabler' and we simply took PUMA into consideration. PUMA serves its function as an 'Enabler', although recently reports regarded it as an 'Activator' [60]. Being an 'Activator' remains obscure because another report did not support that [61]. If PUMA fulfilled both roles, the threshold that triggers apoptosis would dramatically decrease (data not shown). Although Bid is a transcriptional target of p53 and functions as an 'Activator' [6,62] in some cases, it remains un-induced even 20 h after irradiation [63]. If Bid together with PUMA is both induced, the threshold that triggers apoptosis in our model will result in a dramatically 2-fold decrease (data not shown). Bax serves as a member of p53 transcription regime [6,63], and if considered in our model, it provides a resembling decrease in the threshold damage (data not shown). PUMA, Noxa, Bid and Bax, although diverse in their transcriptional kinetics, achieve their goals directly or indirectly by disrupting mitochondria outer membrane. We simply proposed a qualitative model to uncover the intricate regulations in the death decision process, and further suggest that p53 pulses provide a gauge for the severity of genome instability and signal to the Bax activation switch through transcription of whatever 'messengers' (PUMA, Noxa, Bid or Bax) to lead the Bax activation switch to decide cell fate. Parameters were modestly adjusted to capture the intuitive idea of our present model (see Additional file 2, Table S1 for equations and additional file 3, Table S2 for parameters).

The ordinary differential equations in our paper were integrated using ode23s operator in MATLAB (The MathWorks, Natick, MA) platform (version 7.0, Release 14). Simulation programs were written in M files. Bifurcation analysis was performed with AUTO embedded in Oscill8 .

## Authors' contributions

TZS, CC and JC designed the study and coordinated the work. YYW and SZ analyzed parts of the data and refined the pictures. TZS and JC wrote the paper and all authors read and approved the final manuscript. PPS and JC supervised the project.

## Supplementary Material

Additional file 1 **Figure S1. Comparison of p53 dynamics**. **A) **The temporal response of p53 (IR = 20 Gy). The repairing competence of MRN was considered here by multiplying [MRN*] to the right side of Eq. 3. **B) **Time series plot of the model we use in our model. Click here for file

Additional file 2 **Table S1. Model equations for Bax module**. Table S1 describes ordinary equations for Bax activation switch and these equations are derived from our previous model (see main text). Click here for file

Additional file 3 **Table S2. Parameters for Bax activation module**. Table S2 describes parameters for Bax activation module and the parameters are modestly adjusted from our previous work. Click here for file
