# Supplementary figures and images for: Modeling the role of p53 pulses in DNA damage- induced cell death decision
Source: BMC Bioinformatics. 2009 Jun 22;10:190. doi: 10.1186/1471-2105-10-190 (PMC2713228; doi:10.1186/1471-2105-10-190)

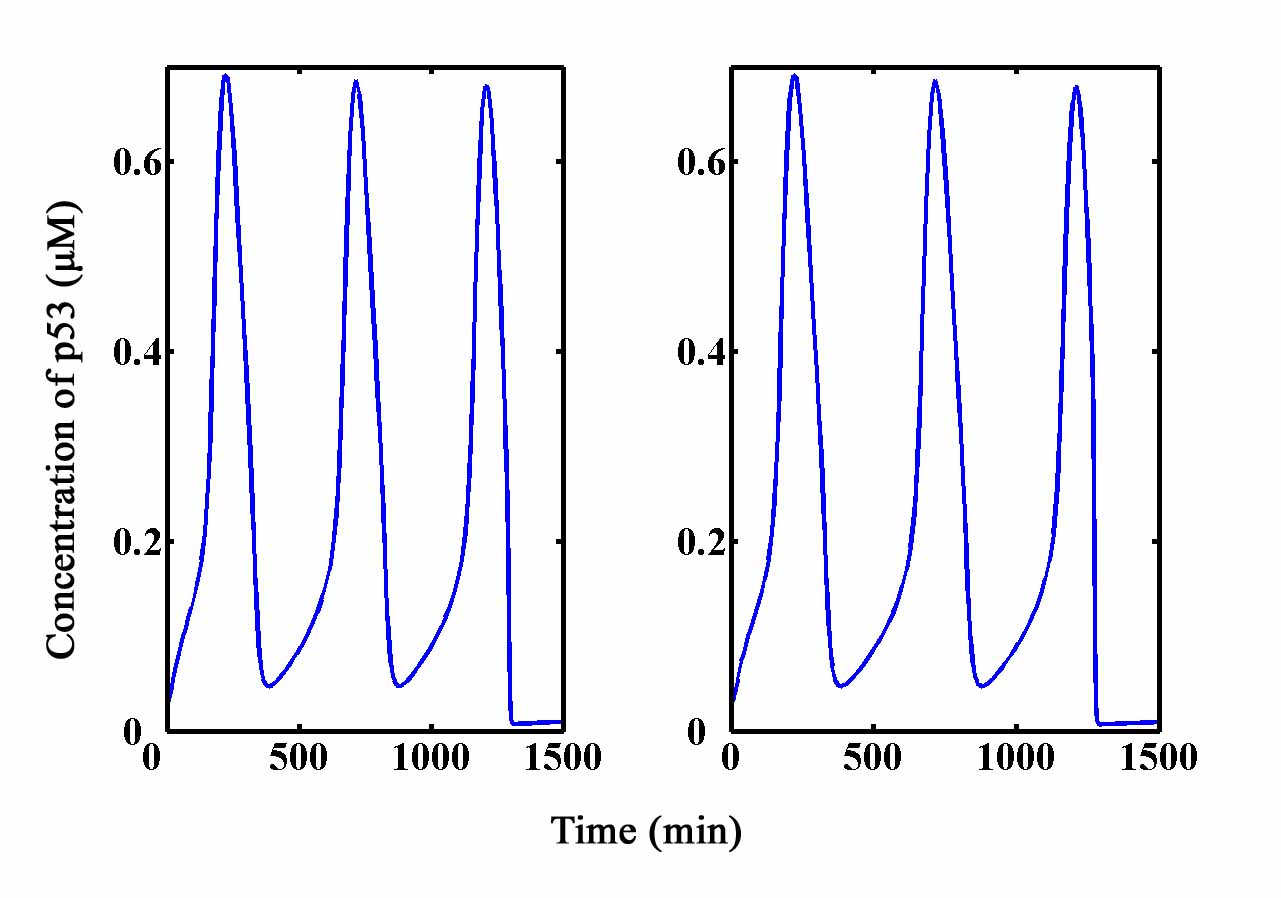

Supplement: Additional file 1 — Figure S1. Comparison of p53 dynamics. A) The temporal response of p53 (IR = 20 Gy). The repairing competence of MRN was considered here by multiplying [MRN*] to the right side of Eq. 3. B) Time series plot of the model we use in our model. [file 1471-2105-10-190-S1.jpeg]
