# Supplementary material for: Modeling the role of p53 pulses in DNA damage- induced cell death decision
Source: BMC Bioinformatics. 2009 Jun 22;10:190. doi: 10.1186/1471-2105-10-190 (PMC2713228; doi:10.1186/1471-2105-10-190)
Supplement: Additional file 2 — Table S1. Model equations for Bax module. Table S1 describes ordinary equations for Bax activation switch and these equations are derived from our previous model (see main text). [file 1471-2105-10-190-S2.pdf]

Table S1: Equations for Bax activation module

$$\frac{d[InBax]}{dt} = p_1 - u_1 \cdot [InBax] - k_{b1} \cdot [InBax] \cdot [Act] - k_{b15} \cdot [InBax] \cdot [AcBax] \quad (8)$$

$$\begin{aligned}\frac{d[AcBax]}{dt} = & -u_5 \cdot [ActBcl2] + k_{b1} \cdot [InBax] \cdot [Act] - k_{b2} \cdot [AcBax] \cdot [Bcl2] \\ & + k_{b3} \cdot [AcBaxBcl2] - k_{b6} \cdot [AcBax] \cdot [ActBcl2] + k_{b7} \cdot [AcBaxBcl2] \cdot [Act] \\ & - k_{b8} \cdot [AcBax] + k_{b13} \cdot [Ena] \cdot [AcBaxBcl2] - k_{b14} \cdot [AcBax] \cdot [EnaBcl2] \\ & - k_{b15} \cdot [InBax] \cdot [AcBax] - 2 \cdot k_{b16} \cdot [AcBax]^2 + 2 \cdot k_{b17} \cdot [MAC]\end{aligned} \quad (9)$$

$$\begin{aligned}\frac{d[Bcl2]}{dt} = & \; p_3 - u_4 \cdot [Bcl2] - k_{b2} \cdot [AcBax] \cdot [Bcl2] + k_{b3} \cdot [AcBaxBcl2] \\ & - k_{b4} \cdot [Act] \cdot [Bcl2] + k_5 \cdot [ActBcl2] - k_{b9} \cdot [Ena] \cdot [Bcl2] + k_{b10} \cdot [EnaBcl2]\end{aligned} \quad (10)$$

$$\begin{aligned}\frac{d[Act]}{dt} = & \; p_2 - u_3 \cdot [Act] - k_{b1} \cdot [InBax] \cdot [Act] - k_{b4} \cdot [Act] \cdot [Bcl2] \\ & + k_{b5} \cdot [ActBcl2] + k_{b6} \cdot [AcBax] \cdot [ActBcl2] - k_{b7} \cdot [AcBaxBcl2] \cdot [Act] \\ & + k_{b11} \cdot [Ena] \cdot [ActBcl2] - k_{b12} \cdot [Act] \cdot [EnaBcl2]\end{aligned} \quad (11)$$

$$\begin{aligned}\frac{d[ActBcl2]}{dt} = & -u_5 \cdot [ActBcl2] + k_{b4} \cdot [Act] \cdot [Bcl2] - k_{b5} \cdot [ActBcl2] \\ & - k_{b6} \cdot [AcBax] \cdot [ActBcl2] + k_{b7} \cdot [AcBaxBcl2] \cdot [Act] \\ & - k_{b11} \cdot [Ena] \cdot [ActBcl2] + k_{b12} \cdot [Act] \cdot [EnaBcl2]\end{aligned} \quad (12)$$

$$\begin{aligned}\frac{d[AcBaxBcl2]}{dt} = & -u_6 \cdot [AcBaxBcl2] + k_{b2} \cdot [AcBax] \cdot [Bcl2] - k_{b3} \cdot [AcBaxBcl2] \\ & + k_{b6} \cdot [AcBax] \cdot [ActBcl2] - k_{b7} \cdot [AcBaxBcl2] \cdot [Act] \\ & - k_{b13} \cdot [Ena] \cdot [AcBaxBcl2] + k_{b14} \cdot [AcBax] \cdot [EnaBcl2]\end{aligned} \quad (13)$$

$$\begin{aligned}\frac{d[Ena]}{dt} = & \; p_4 - u_7 \cdot [Ena] - k_{b9} \cdot [Ena] \cdot [Bcl2] + k_{b10} \cdot [EnaBcl2] \\ & - k_{b11} \cdot [Ena] \cdot [ActBcl2] + k_{b12} \cdot [Act] \cdot [EnaBcl2] \\ & - k_{b13} \cdot [Ena] \cdot [AcBaxBcl2] + k_{b14} \cdot [AcBax] \cdot [EnaBcl2] \\ & + k_{puma} \cdot [p53]^{np} / (K_f^{\,np} + [p53]^{np})\end{aligned} \quad (14)$$

$$\begin{aligned}\frac{d[EnaBcl2]}{dt} = & -u_8 \cdot [EnaBcl2] + k_{b9} \cdot [Ena] \cdot [Bcl2] - k_{b10} \cdot [EnaBcl2] \\ & + k_{b11} \cdot [Ena] \cdot [ActBcl2] - k_{b12} \cdot [Act] \cdot [EnaBcl2] \\ & + k_{b13} \cdot [Ena] \cdot [AcBaxBcl2] - k_{b14} \cdot [AcBax] \cdot [EnaBcl2]\end{aligned} \quad (15)$$

$$\frac{d[MAC]}{dt} = -u_9 \cdot [MAC] + k_{b15} \cdot [InBax] \cdot [AcBax] + k_{b16} \cdot [AcBax]^2 - k_{b17} \cdot [MAC] \quad (16)$$
