# Supplementary material for: Modeling the role of p53 pulses in DNA damage- induced cell death decision
Source: BMC Bioinformatics. 2009 Jun 22;10:190. doi: 10.1186/1471-2105-10-190 (PMC2713228; doi:10.1186/1471-2105-10-190)
Supplement: Additional file 3 — Table S2. Parameters for Bax activation module. Table S2 describes parameters for Bax activation module and the parameters are modestly adjusted from our previous work. [file 1471-2105-10-190-S3.pdf]

Table S2: Parameters for Bax activation module

$k_{b1} = 0.1\ \mu M^{-1} \cdot min^{-1}$	$p_1 = 0.02\ \mu M \cdot min^{-1}$
$k_{b2} = 1 \mu M^{-1} \cdot min^{-1}$	$p_2 = 0.002\ \mu M \cdot min^{-1}$
$k_{b3} = 0.001\ min^{-1}$	$p_3 = 0.006\ \mu M \cdot min^{-1}$
$k_{b4} = 10\ \mu M^{-1} \cdot min^{-1}$	$p_4 = 0.002\ \mu M \cdot min^{-1}$
$k_{b5} = 0.06\ min^{-1}$	$u_1 = 0.03\ min^{-1}$
$k_{b6} = 0.5\ \mu M^{-1} \cdot min^{-1}$	$u_2 = 0.002\ min^{-1}$
$k_{b7} = 0.01\ \mu M^{-1} \cdot min^{-1}$	$u_3 = 0.01\ min^{-1}$
$k_{b8} = 0.001\ min^{-1}$	$u_4 = 0.002\ min^{-1}$
$k_{b9} = 0.1\ \mu M^{-1} \cdot min^{-1}$	$u_5 = 0.002\ min^{-1}$
$k_{b10} = 0.001\ min^{-1}$	$u_6 = 0.01\ min^{-1}$
$k_{b11} = 0.5\ \mu M^{-1} \cdot min^{-1}$	$u_7 = 0.001\ min^{-1}$
$k_{b12} = 0.05\ \mu M^{-1} \cdot min^{-1}$	$u_8 = 0.005\ min^{-1}$
$k_{b13} = 10\ \mu M^{-1} \cdot min^{-1}$	$u_9 = 0.01\ min^{-1}$
$k_{b14} = 0.5\ \mu M^{-1} \cdot min^{-1}$	$k_{puma} = 0.005\ \mu M \cdot min^{-1}$
$k_{b15} = 0.2\ \mu M^{-1} \cdot min^{-1}$	$K_f = 0.5\ \mu M$
$k_{b16} = 0.2\ \mu M^{-1} \cdot min^{-1}$	
$k_{b17} = 0.01\ min^{-1}$	

Parameters in Bax activation module are adopted from our previous work within one order of magnitude [28].
